# Paradoxes of breast cancer incidence and mortality in two corners of Europe

**DOI:** 10.1186/s12885-022-10243-w

**Published:** 2022-11-02

**Authors:** Mikaela Roginski, Dimitra Sifaki-Pistolla, Andreas Stomby, Georgia Velivasaki, Tomas Faresjö, Christos Lionis, Åshild Faresjö

**Affiliations:** 1 Department of Health, Medicine and Caring Sciences, Linköping University, SE-581 83 Linköping, Sweden; 2 Clinic of Social and Family Medicine, Faculty of Medicine, University of Crete, Heraklion, Greece

**Keywords:** Breast cancer, Comparative study, Incidence, Mortality, Register data

## Abstract

**Background:**

Breast cancer incidence is rising globally, while mortality rates show a geographical heterogenous pattern. Early detection and treatment have been proven to have a profound impact on breast cancer prognosis. The aim of his study was to compare breast cancer incidence, mortality, and survival rates in two contrasting corners of Europe, Sweden and Crete, to better understand cancer determinants with focus on disease burden and sociocultural factors.

**Methods:**

Breast cancer data from Sweden and Crete was derived from registries. Incidence and mortality were expressed as Age-Standardized Incidence Rates (ASIR), Age-Standardized Mortality Rates (ASMR).

**Findings:**

Breast cancer incidence has for decades risen in Sweden and on Crete. In 2019, ASIR was 217.5 in Sweden and 58.9 on Crete, (*p* < 0.001). Mortality rates showed opposite trends. ASMR in Sweden was reduced from 25.5 to 16.8 (2005-2019) while on Crete, ASMR increased from 22.1 to 25.3. A successive rise in survival rate in Sweden with a 5-year survival rate of 92% since 2015, but a converse development on Crete with 85% 5-year survival rate the same year.

**Interpretation:**

The incidence of breast cancer is slowly rising in both studied regions, but mortality increases on Crete in contrast to Sweden with sinking mortality rates. The interpretation of these findings is that differences in health care systems and health policies including differences in early detection like screening programs and early treatment, as well as sociocultural factors in the two countries might play an important role on the differences found in breast cancer burden.

## Background

The frequency of breast cancer rises in almost every region globally and in all age groups [1]. Recent data shows for the first time that breast cancer is the most frequently diagnosed cancer in women worldwide [2]. The total number of deaths from breast cancer constituted 15% of all cancer-related deaths globally in 2018 [3]. For females, breast cancer is the deadliest form of cancer disease, and the global burden of breast cancer increases in both premenopausal and postmenopausal age groups [3, 4]. In Sweden, breast cancer currently accounts for 30% of all cancer cases of women [5] and the corresponding data for Greece is 27.5% [6]. The implication of breast cancer disease also includes aspects of psychosocial health and quality of life [7].

Radiation is the most established risk factor for breast cancer, generating DNA-damage in breast tissue [8]. However, around 5-10% of all breast cancer cases are linked to heredity [9]. General underlying mechanisms of breast cancer have been coupled to the estrogen pathway, i.e., long-term exposure of female sex-hormones due to factors such as early menarche and late menopause, nullipara, high age at first child etc. [10], but also the increase in life expectancy per se [2]. Suggested potential non-estrogenic risk factors with various degree of scientific evidence are alcohol consumption, physical inactivity [11], and psychosocial factors such as social stress [12, 13]. Also, in a salutogenic perspective, dietary factors such as the Mediterranean diet has been extensively studied, although with shifting results [14]. Earlier comparative studies between Sweden and Greece from our group have shown differences in morbidity, both from cancer and cardiovascular disease [15]. There are differences in the health care systems and structures but also in health care utilization, drug prescription, perceived health, health literacy and risk perceptions between the populations of these two regions in Europe [16, 17].

According to current knowledge, early detection with early, effective treatment are the fundamental factors improving breast cancer prognosis [2, 18]. In Sweden, a national screening program for early detection of breast cancer is used since the 1980’s, and since 2015, a national streamlined standardized course of investigation is implemented to reduce time-intervals during the diagnostical process [19]. In Greece, a national screening program is also available but not fully implemented in practice till today, since there is no systematic approach in monitoring, reminding or following-up individuals at risk. Breast cancer screening is considered opportunistic [20].

With an explorative, epidemiological, and comparative view, we will in this study scrutinize trends in breast cancer data on Crete and in Sweden over the years. Cancer statistics of this epidemiological kind has previously not been available for Crete, only hospitalization data [15]. *The aim* of this study was to analyze and compare breast cancer incidence, mortality, and survival rates in Sweden and on Crete, Greece. By contrasting two corners of Europe representing the Scandinavian countries and the Mediterranean, we anticipate that such comparative view could assist both planners for health care services and clinical researchers to better understand cancer determinants with focus on cancer burden and sociocultural factors in various European settings, a topic on the epicenter of the current health policy discussion.

## Materials and methods

### Study design and data material

The study is an epidemiological, register study of national and regional data. ASIR corresponds to age-standardized incidence rate (per 100.000 inhabitants) and ASMR to age-standardized mortality rate (per 100.000 inhabitants). The incidence was calculated by a direct standardization approach according to the WHO/European standard population. The Swedish data collection was extracted from open registries of Swedish National Board of Health and Welfare (for ASIR) and NORDCAN (for ASMR), their collaborative database with WHO with age-standardization according to International Cancer Survival Standard [21]. The data included in this study constitutes of new breast cancer cases (for ASIR) among women of the total Swedish population. Age interval was 20-74 years, diagnosis ICD C50.X (in situ excluded) [21]. The Cretan population-based data derives from the regional cancer registry of Crete [22, 23]. In this study, the 5- and 10- year survival rate, respectively, are stated as a rolling average of three consecutive years (− 1-year-1+). The estimates of breast cancer survival, i.e. 5- and 10-years survival, are calculations of prognosis, based on the last 5 or 10 years, respectively. The clarification of breast cancer is the same for Sweden and Crete, and the methodological processing of data has been equivalent for both countries.

The coverage of the Swedish national cancer register is extremely high, approximately around 99%, and the survival estimates are even higher [24]. The Cretan breast cancer data used in this study derives from The Cancer Registry of Crete and had 98.5% completeness, 96.4% reliability, 100% timeliness and 99% continuity. The two studied populations of two corners of Europe should be studied in relation to corresponding local health care setting. In Table 1, descriptive characteristics of the breast cancer care system are illustrated.

**Table 1:** Descriptive characteristics of breast cancer care systems in Crete, Greece, and for Sweden

	GRE	SWE
Availability of national screening program of breast cancer	Not implemented in practice (low levels of compliance and no reminders from the healthcare systems/clinics etc).	Yes, national mammography screening every second year for all women 40-74 years.
Streamlined, standardized course of investigation of breast cancer	Not comprehensive	Yes
Claim of remittance^a^	No	No
Specialized breast cancer units/clinics	Yes	Yes
Cost for health care visit	Yes, general health insurance for all Cretan citizens covering most of the cost.	Yes, a general health insurance for all Swedish citizens covering most of the cost.

^a^Claim of remittance from another health care unit, in contrast to self-remittance

### Statistical analyses

We calculated mean ASIR and mean ASMR per 100.000 population. The calculations of *p*-values for the Swedish and the Cretan incidence and mortality data were based on aggregated data per 100.000 inhabitants, which means these calculations are approximations. *P*-values were overall estimated by z test one sample, since there was no information on population variance and the standard deviation was used in this model. Specifically, the z test one sample was performed to compare the ASIR, ASMR, 5- and 10-year survival rates within Sweden and Crete, respectively. For comparisons of the rates between the two countries, the z test two samples were used.

## Results

In 2019, ASIR was 58.9 in Crete and 217.5 in Sweden, varying statistically significant between the two regions (*p* < 0.001). On the contrary, ASMR was 25.5 in Crete and 16.8 in Sweden (*p* = 0.04).

**Fig. 1:**
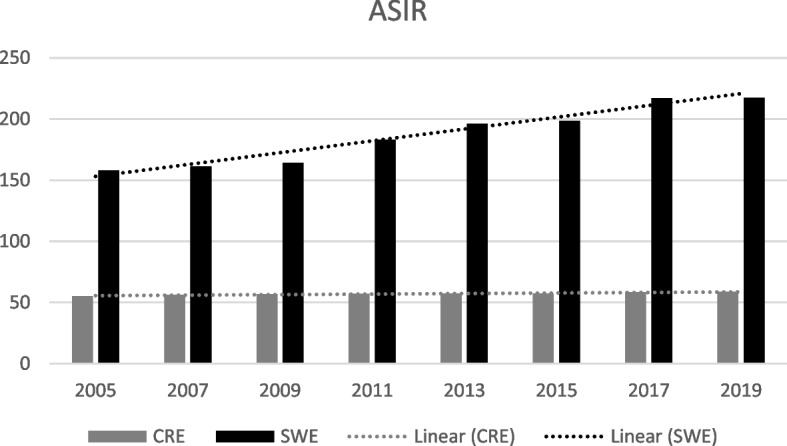
ASIR for breast cancer and trends on Crete compared to Sweden over the last decades

**Fig. 2:**
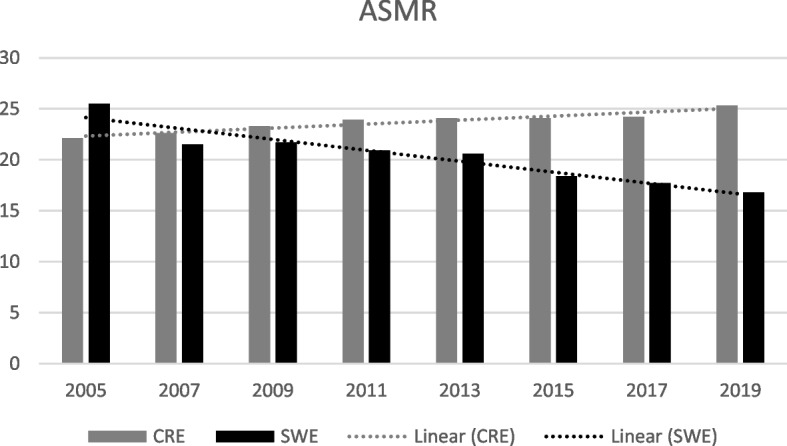
ASMR for breast cancer and trends on Crete compared to Sweden over the last decades

Over the last 15 years, the incidence rate of breast cancer in Sweden has successively tendered to rise from ASIR 158.1 in 2005 to 217.5 in 2019 (*p* = 0.12). The mortality rate, in turn, has shown a falling trend over the years, ASMR being 25.5 in 2005 and 16.8 in 2019 (*p* = 0.001). On Crete, ASIR is on a lower level in general compared to Sweden (*p* < 0.001), presented in Fig. 1, but similarly display a rising trend during the last 1.5 decade, with ASIR 55.3 in 2005 and 58.9 in 2019 (*p* = 0.002). The Cretan mortality rate, however, show a deviant pattern with a rising ASMR from 22.1 in 2005 to 25.3 in 2019 (*p* = 0.003) as seen in Fig. 2. Before 2007, Crete had an ASMR 22.1, while in Sweden the ASMR was 25.5 (*p* = 0.01). Nevertheless, after the year 2005, the Cretan mortality rates show a successively rising trend, while Swedish mortality rates have been descending.

**Fig. 3:**
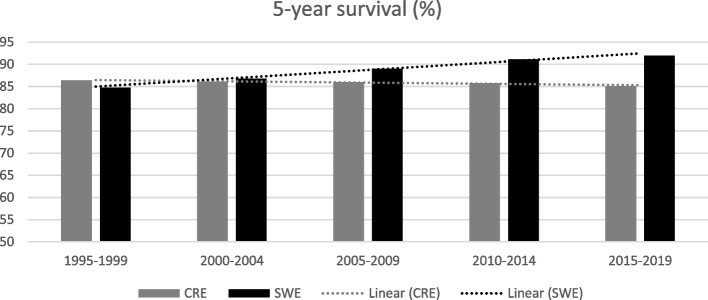
5-year survival (%) for breast cancer and trends on Crete compared to Sweden during the decades around the millennium

**Fig. 4:**
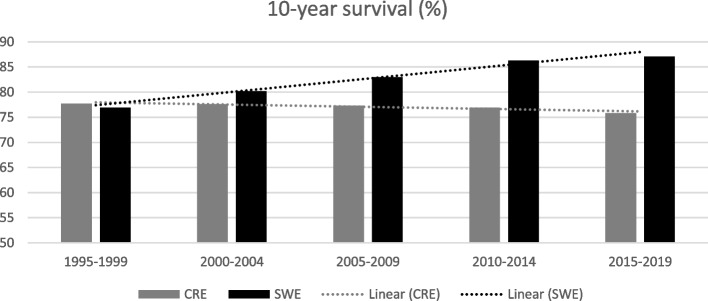
10-year survival (%) for breast cancer and trends on Crete compared to Sweden during the decades around the millennium

As regards survival, increasing trends of survival rates has been seen in Sweden in contrast to decreasing trends on Crete, but this difference is not statistically significant (*p* = 0.98). Specifically, 5-year survival in Sweden from 1995 to 2019 ranged from 84.8 to 92.0% (*p* = 0.02), with a 5-year survival > 90% during the last decade, illustrated in Fig. 3. Conversely, 5-year survival in Crete, ranged from 86.4 to 85.1% during the same period. Regarding the 10-year survival rate, presented in Fig. 4, Swedish data ranged from 76.9% in 1995 to 87.1% in 2019 (*p* = 0.02). Over time, regional 10-year survival data in Crete ranged from 77.1 to 75.8% for the same period (*p* = 0.04).

## Discussion

The main findings in this study show a rising breast cancer incidence in both Crete and Sweden, however, a paradox is that mortality rate in Crete has increased last decade, while in Sweden, breast cancer mortality decreased. This is a striking difference in the pattern of breast cancer burden for populations of these two regions on the European continent. Regarding survival rates, trends of survival in Sweden have increased over time in contrast to a lack of improvement - and even small decline *-* in survival on Crete since 1995.

The findings in this study must be seen against a multifactorial background where both health care systems as well as life-style factors and salutogenic factors might play important roles. Breast cancer mortality and survival are tightly related. It is generally stated, that one of the most important, modifiable, known parameters with influence on prognosis is early detection as a prerequisite for early, effective treatment with medical drugs and interventions that is already known to work [18].

In many European countries like the Scandinavian countries, Germany, Italy, Spain, Poland and others, there has been a marked reduction in the breast cancer mortality rates (between 8 and 19%) for the last years. This reduction has in general been attributed to earlier detection and improved treatment [11]. However, in Greece, a national mammography screening program has yet to be fully implemented [20]. There is yet no nationally formulated strategy for early detection of breast cancer in Greece.

Several factors are involved in the process leading to diagnosis, from the individuals own detection and insight at the debut of symptoms, to the availability and utilization of local health care. Mammographic screening on a population level is an established method to approach early detection of breastcancer before clinical symptoms occur [25]. In Sweden, 60% of all breast cancer cases are detected through the national screening program for women between 40 and 74 years of age [21, 26]. In countries with a tradition of mammography screening of breast cancer, an increase in incidence rates and a decrease in mortality rates have been evident for decades [27]. This may point to an increased incidence related to improved diagnostics but may also stem from risk of overdiagnostic. Nevertheless, potential overdiagnostic may complicate the interpretation of epidemiological health statistics. In a metaanalysis from 2012, the overdiagnostics was estimated to be 11% during lifetime for a woman invited to the screening program, and 19% during the specific time-period of inclusion in the screening program [28]. However, in many countries the incidence rise began before the mammography screening programs were implemented, also seen in countries who introduced screening programs relatively late [27]. In different populational settings, different challenges are distinguished related to features of the population [29]. The mammography screening of women, at least for the age-group 50-69 years, is one way to significantly reduce mortality rates of breast cancer [25], but mammography screening is also under an ongoing debate [30].

Another way into early diagnosis, is to make the diagnostical process per se more effective. In Sweden, a time-regulated, standardized, health care process is used when symptoms leading to high suspicion of breast cancer. Through this process, from the referral of the patient to a specialized oncological hospital unit, the individual has priority to required examinations and the time space between examinations and clinical consultations are strictly time-regulated [19].

The structure of the health care systems and the availability of health care differ between the studied regions, but there are also similarities, as illustrated in Table 1. In a time perspective, the Greek economy experienced a long period of recession during the period 2007-2015, with retrenchments in health care [31]. After the Greek recession, factors like quality of treatment as well as funding and access to health care have been stated a particular challenge of the society [32]. Interestingly, these years of economic recession overlaps the period of declination of the 10-year survival in Greece.

Sociocultural aspects with discrepancies in perceptions of health and disease may be factors of concern regarding general health literacy among the populations studied [17, 33]. In a study of Cretan women, several reasons for not using mammography were identified, like poor knowledge of the benefits of mammography screening, lack of physician recommendation, costs, embarrassment, fear of pain during the procedure and fear of a serious diagnosis [33]. Sociocultural aspects regarding doctor-patient relations and areas associated with high personal integrity, like the clinical investigations of the female breast, might play a role in patient-compliance. Communicative factors, such as language barriers, might also be factors of concern.

The strength of the study is that both the Swedish and Cretan data derives from solid and reliable registers. In Sweden, there is a historical tradition of registries with one of the world’s oldest cancer registries, started in 1958, with a national coverage [21]. The reporting of all new cancer cases is obligatory by law in Sweden, both from physician in charge as well as the responsible unit for pathological and cytological laboratory. In this way, the Swedish Cancer registry cover approximately around 99% of all cases and in 2015 an investigation showed that 100% of the reported cases were verified with cytology or histology, pointing to a valid and accurate measure [24]. National cancer registry is not yet available for the whole country of Greece, but for the region of Crete. The Cancer Registry of Crete has reached high numbers of data quality by following the European Network of Cancer Registries (ENCR) quality standards, which evaluate four dimensions (i.e., completeness, reliability, timeliness, and continuity). A limitation in this study is that the Greek data is only available from a specific region of the country, the island of Crete. The Swedish screening tool might introduce some bias. According to Swedish National Quality Registry of Breast Cancer (NKBC), every tumor of the breast found through screening is treated even if the knowledge is scarce about how the tumor would have developed with time if left untreated [27]. This perception may have influence on the incidence as well as the mortality data, possibly contributing to higher number of cases found, and consequently lower mortality rate in Sweden.

In conclusion, this study shows a contrasting pattern of breast cancer burden between two corners of the same European continent. Although the incidence is slowly rising in both regions, the mortality is increasing on Crete in contrast to Sweden where the mortality trend is decreasing. The findings further reveal a rising survival rate in Swedish breast cancer patients, while the survival trends on Crete are falling. An interpretation of these findings is that differences in health care systems and health policies as well as sociocultural factors between the two countries might play an important role on the outcome of breast cancer. The findings also indicate the need for a national breast cancer strategy in Greece, possibly with a national screening program and a streamlined, standardized course of investigation to improve early diagnostical processes and early treatment.

## Data Availability

The Swedish data is official and available at NORDCAN/WHO and the Swedish National Board of Health and Welfare. The Cretan datasets generated during and analyzed during the current study are not publicly available due to Greek official regulations but are available from the corresponding author on reasonable request.
